# Criteria for Reporting and Evaluating ecotoxicity Data (CRED): comparison and perception of the Klimisch and CRED methods for evaluating reliability and relevance of ecotoxicity studies

**DOI:** 10.1186/s12302-016-0073-x

**Published:** 2016-02-29

**Authors:** Robert Kase, Muris Korkaric, Inge Werner, Marlene Ågerstrand

**Affiliations:** 1 Swiss Centre for Applied Ecotoxicology, EAWAG-EPFL, 8600 Dübendorf, Switzerland; 2 Department of Environmental Toxicology, Swiss Federal Institute of Aquatic Science and Technology (Eawag), 8600 Dübendorf, Switzerland; 3 Department of Environmental Science and Analytical Chemistry (ACES), Stockholm University, 106 91 Stockholm, Sweden

**Keywords:** Reliability evaluation, Relevance evaluation, Klimisch method, Hazard/risk assessment, Water quality criteria, Ecotoxicology

## Abstract

**Background:**

The regulatory evaluation of ecotoxicity studies for environmental risk and/or hazard assessment of chemicals is often performed using the method established by Klimisch and colleagues in 1997. The method was, at that time, an important step toward improved evaluation of study reliability, but lately it has been criticized for lack of detail and guidance, and for not ensuring sufficient consistency among risk assessors.

**Results:**

A new evaluation method was thus developed: Criteria for Reporting and Evaluating ecotoxicity Data (CRED). The CRED evaluation method aims at strengthening consistency and transparency of hazard and risk assessment of chemicals by providing criteria and guidance for reliability and relevance evaluation of aquatic ecotoxicity studies. A two-phased ring test was conducted to compare and characterize the differences between the CRED and Klimisch evaluation methods. A total of 75 risk assessors from 12 countries participated. Results show that the CRED evaluation method provides a more detailed and transparent evaluation of reliability and relevance than the Klimisch method. Ring test participants perceived it to be less dependent on expert judgement, more accurate and consistent, and practical regarding the use of criteria and time needed for performing an evaluation.

**Conclusions:**

We conclude that the CRED evaluation method is a suitable replacement for the Klimisch method, and that its use may contribute to an improved harmonization of hazard and risk assessments of chemicals across different regulatory frameworks.

**Electronic supplementary material:**

The online version of this article (doi:10.1186/s12302-016-0073-x) contains supplementary material, which is available to authorized users.

## Background

The availability of reliable and relevant ecotoxicity data is a prerequisite for hazard and risk assessment of chemicals in regulatory frameworks, e.g., marketing authorization of plant production products [1], biocides [2], and human pharmaceuticals [3], and within the Water Framework Directive (WFD) [4] and REACH legislations [5]. In most of these frameworks, ecotoxicity studies used for risk assessment must undergo an evaluation of reliability and relevance, as described in detail in the REACH guidance. REACH defines reliability as “the inherent quality of a test report or publication relating to preferably standardized methodology and the way the experimental procedure and results are described to give evidence of the clarity and plausibility of the findings.” Relevance is defined as “the extent to which data and tests are appropriate for a particular hazard identification or risk characterisation” [5].

Similarly, the United States Environmental Protection Agency (US EPA) has published guidelines for screening, reviewing, and using published open literature toxicity data in ecological risk assessments [6]; however, these lack detailed guidance regarding relevance evaluation. Available European guidance documents, e.g., the REACH guidance document, the European Commission’s Technical Guidance Document (TGD) [7], and WFD [8], do not provide detailed information on how to evaluate reliability and relevance of a study. This lack of guidance causes study evaluations to depend strongly on expert judgement, which in turn can result in discrepancies among assessments and disagreements among risk assessors on whether or not a study can be used for regulatory purposes. Evaluation criteria and minimum reporting requirements for scientific test results should ensure that regulatory decisions are taken on a thorough and verifiable basis [9]. In this context, a need for applying robust and science-based principles in ecotoxicology has been stipulated [10, 11].

Currently, the evaluation method proposed by Klimisch et al. in 1997 [12] forms the backbone of many regulatory procedures where reliability of ecotoxicity studies needs to be determined. The Klimisch method provides a system where ecotoxicity studies categorized as either “reliable without restrictions” or “reliable with restrictions” are generally considered adequate for use in environmental hazard and risk assessments, while those categorized as “not reliable” are not accepted for regulatory use. Studies categorized as “not assignable” lack the detailed information needed to evaluate reliability. Depending on the framework and the reasons for lower reliability, studies classified as “not reliable” and “not assignable” may be used as supporting information. Even though the Klimisch method is widely used, some shortcomings have become evident and the method has been increasingly subject to criticism. Recent studies have demonstrated that the Klimisch method does not guarantee consistent evaluation results among different risk assessors [13, 14]. It provides only limited criteria for reliability evaluation and no specific guidance for relevance evaluation [15, 16]. Insufficient guidance increases the inconsistency of evaluation results. As a result of this, an ecotoxicity study may be categorized as “reliable with restrictions” by one risk assessor and as “not reliable” by another, leading to disagreement regarding the inclusion of the study in a data set used for hazard or risk assessment. Such inconsistent evaluation results can directly influence the outcome of a hazard or risk assessment for a specific chemical, which in turn may result e.g., in unnecessary risk mitigation measures, or underestimated risks for the environment. It is therefore essential that all available studies are evaluated using a science-based method that promotes consistency and transparency.

The Klimisch method favors studies performed according to Good Laboratory Practice (GLP) [13, 16, 17] and validated ecotoxicity protocols, such as those provided by the Organisation for Economic Co-operation and Development (OECD) and the US EPA. This can lead to a situation where risk assessors automatically categorize such studies as reliable without restrictions, even if obvious flaws (e.g., control mortality above accepted level, selection of non-relevant endpoints) exist. The preference for GLP studies has led to marketing authorization dossiers which rely almost exclusively on contract laboratory data provided by the registrants, while excluding peer-reviewed studies from the scientific literature. This has been openly criticized [18], and several regulatory frameworks [1, 5, 6, 8] have recently recommended to take all available information into account. This is important since hazard and risk assessments and the derivation of environmental quality criteria (EQC) or standards (EQS) for individual chemicals often suffer from limited data availability. In addition, the inclusion of more peer-reviewed studies offers the potential to save resources, both from an economic and ethical (number of animals used) point of view.

Multiple evaluation methods are available to risk assessors [6, 21–27]; however, most focus exclusively on the evaluation of reliability with the exception of those provided by Ågerstrand et al. [15] and Beronius et al. [24], while the equally important relevance aspects of a study are not considered. A comparison of four methods for reliability evaluation of ecotoxicity studies showed that choice of method affected the outcome [13]. In addition, a review of methods used to evaluate toxicity studies concluded that only five of 30 methods investigated had been rigorously tested by risk assessors in order to evaluate their applicability [28]. Other attempts to score study reliability were developed by industry and other institutions [23]; however, to our knowledge they—like most other methods—were not implemented in regulatory guidance documents. A method that provides increased transparency and consistency in evaluations is therefore needed to reduce uncertainties regarding the assessment of (key) ecotoxicity studies and provide more harmonized hazard and risk assessments.

The Criteria for Reporting and Evaluating ecotoxicity Data (CRED) project resulted from a 2012 initiative focused on the need for improvement of the Klimisch method [16]. The project aims at strengthening the transparency, efficiency, and robustness of environmental hazard and risk assessments of chemicals, and increasing the utilization of peer-reviewed ecotoxicity studies for substance evaluations through improved reporting. To this end, the CRED evaluation method and the CRED reporting recommendations were developed. The CRED evaluation method provides reliability and relevance evaluation criteria and detailed guidance material [19, 20]. These tools can be used for prospective and retrospective hazard and risk assessment of chemicals, as well as in the peer-review process.

This publication presents results of a ring test aimed at comparing the CRED and Klimisch evaluation methods with regard to study categorization, consistency of evaluations, the risk assessors’ perception of the methods, and practical aspects such as time requirements and the use of proposed evaluation criteria. It was our aim to demonstrate that the increased guidance provided by the CRED evaluation method would reduce inconsistency and increase transparency of study evaluations while being practical in use.

## Methods

### Development of the CRED evaluation method

The CRED evaluation method is based on OECD ecotoxicity test guidelines (e.g., OECD guidelines 201, 210 and 211), existing evaluation methods (e.g., [15, 25]), as well as practical expertise in evaluating studies for regulatory purposes. The method was presented and discussed at several expert meetings including the Society of Environmental Toxicology and Chemistry (SETAC) Global Environmental Risk Assessment Advisory Group, and the SETAC Global Pharmaceutical Advisory Group. Feedback from these meetings and ring test participants, as well as results of the ring test, was incorporated into the development of the final version of the CRED evaluation method [19]. General characteristics of the Klimisch and CRED evaluation methods are provided in Table 1.

**Table 1:** General characteristics of the Klimisch and the final CRED evaluation methods

Characteristics	Klimisch	CRED
Data type	Toxicity and ecotoxicity	Aquatic ecotoxicity
Number of reliability criteria	12–14 (ecotoxicity)	Evaluating 20 (reporting 50)
Number of relevance criteria	0	13
Number of OECD reporting criteria included^a^	14 (of 37)	37 (of 37)
Additional guidance	No	Yes
How to summarize the evaluation	Qualitative for reliability	Qualitative for reliability and relevance

^a^According to [13]

### Ring test

The ring test was performed in two phases. In phase I (November–December 2012), each participant evaluated reliability and relevance of two out of eight ecotoxicity studies (see below and Table 2) using the Klimisch method. In phase II (March–April 2013), each participant evaluated two different studies from the same set of eight publications according to the CRED evaluation method: It is important to note that the ring test was not performed with the final CRED method [19] but with a draft version of this method (Additional file 1: part A, B). However, differences between these two versions are small (details in Additional file 1: part C Table C1). Studies were assigned based on each participant’s area of expertise, and each study was evaluated by different participants in phases I and II with no overlap within institutes to ensure independent study evaluation.

**Table 2:** List of ecotoxicity studies evaluated in the ring test

Study	Test organism	Taxonomic group					Tested substance	Chemical substance classes					Evaluated endpoint	Peer reviewed	GLP study	References
		Cyanobacteria	Algae	Higher plant	Crustacea	Fish		Industrial chemical	Biocide	Plant protection product	Pharmaceutical	Steroidal estrogens				
A	*Daphnia magna*				X		Deltamethrin			X			EC_50_ 48 h Immobilization	X		[29]
B	*Lemna minor*			X			Erythromycin				X		NOEC 7 days growth	X		[30]
C^a^	*Synechococcus leopoldensis*	X					Erythromycin				X		NOEC 144 h growth	X		[31]
D	*Scenedesmus subspicatus*		X				Deltamethrin			X			NOEC 72 h growth	X		[32]
E^a^	*Danio rerio*					X	Estrone					X	NOEC 40 days sex ratio		X	[33]
F^a^	*Danio rerio*					X	Estrone					X	NOEC 40 days sex ratio	X		[34]
G^a^	*Oncorhynchus mykiss*					X	Nonylphenol	X					NOEC 60 days hatching success	X		[35]
H^a^	*Myriophyllum spicatum*			X			Cybutryne		X				NOEC 14 days growth	X		[36]

^a^Under discussion as a key study for EQS derivation

Since the relevance of a study can vary depending on the regulatory context, risk assessors were informed prior to the evaluation to assume that the studies should be evaluated for their potential use in derivation of EQC within the Water Framework Directive, where all endpoints with known population relevant effects are accepted [4]. Each participant summarized the reliability evaluation into one of the categories established by Klimisch et al. [1]: R1 = Reliable without restrictions, R2 = Reliable with restrictions, R3 = Not reliable, and R4 = Not assignable. Relevance was summarized using the following categories: C1 = Relevant without restrictions, C2 = Relevant with restrictions, and C3 = Not relevant. It should be noted that Klimisch [12] did not suggest any categories for relevance.

Following the evaluation in phases I and II, risk assessors completed a questionnaire (Additional file 1: part A, B) in which they were asked to report their experience using the methods, and their perception of uncertainty regarding the resulting reliability and relevance assessments, and to list evaluation criteria they found to be missing. Based on a consistency analysis for each criterion following phase II, the wording of the CRED evaluation criteria was optimized if consistency (see consistency analysis below) was below 50 %. In addition, criteria identified as missing in these questionnaires were included in the final CRED evaluation method [19, 20], resulting in 20 (instead of 19) reliability criteria and 13 (instead of 11) relevance criteria (SI Table C1). In the draft method, 14 of 19 reliability criteria and 9 of 11 relevance criteria were designated as critical; however, participants explicitly avoided the use of critical criteria and voiced their preference for expert judgement, because a specific criterion may be critical for one test design but not for another. Moreover, the relevance category C4 (=Not assignable) was added to the final CRED evaluation method.

### Ring test participants

Announcements of the ring test were made via email, at the annual meeting of SETAC Europe in 2012 and at several international regulatory meetings. The 75 ring test participants came from 12 countries and 35 organizations. They represented 9 regulatory agencies, 17 consulting companies and advisory groups, and 9 industry and stakeholder organizations. Participants from regulatory agencies were from the following countries: Canada, Denmark, Germany, France, the Netherlands, Sweden, the United Kingdom, and the USA. Regulators from the European Chemicals Agency (ECHA) and other international expert groups, and members of the Multilateral Meeting, where EU risk assessors meet to harmonize national EQC proposals, also joined the exercise. In addition, the following institutions, consulting companies, and advisory groups participated: CEFAS (United Kingdom), CEHTRA (France), CERI (Japan), Deltares (The Netherlands), DHI (Singapore), ECT (Germany), Eurofins AG (Switzerland), GAB Consult (Germany), ITEM (Germany), SETAC Pharmaceutical Advisory Group (PAG), SETAC Global Ecological Risk Assessment Group (ERAAG), Swiss Centre for Applied Ecotoxicology Eawag-EPFL (Switzerland), TSGE (United Kingdom), and wca (United Kingdom). The following industry and stakeholder organizations participated: Astrazeneca (United Kingdom), Bayer (Germany), BASF (Germany), Givaudan International SA (Switzerland), Golder Associates Inc. (United States of America), ECETOC (EU), Harlan (Switzerland), Monsanto Europe (Belgium), and Pfizer (United States of America).

Of a total of 75 participants, 62 participated in phase I (where the Klimisch method was used) and 54 in phase II (where the CRED evaluation method was used), with 76 % of participants taking part in both phases of the ring test. More than 80 % of the participants in phase I had previously used the Klimisch method. The majority of the participants (58 % in phase I and 62 % in phase II) had more than 5 years of experience in performing ecotoxicity study evaluations. The distribution of years of experience among participants in phase I and phase II was similar: 9 and 8 % with 0–1 year, 6 and 10 % with 1–2 years, 27 and 20 % with 2–5 years, 14 and 15 % with 5–10 years, and 44 and 47 % with above 10 years of experience in phase I and II, respectively.

### Selection of ecotoxicity studies to be evaluated

Eight studies were evaluated in the ring test. Seven of these were published in peer-reviewed journals, and one was an industry study report from a contract laboratory (Table 2). Studies were selected by the coordinators of the ring test (who did not participate) to cover different taxonomic groups (cyanobacteria, algae, higher plants, crustacean, and fish), test designs (acute and long-term), and chemical substance classes. Five studies were potential key studies for derivation of EQC proposals in national dossiers. Toxicity endpoints evaluated were biomass, hatching success, sex ratio, growth, and immobilization. It should be noted that the eight studies represent a small selection, which cannot be representative of the broad range of ecotoxicity studies that exist. Based on previous evaluations they were expected to be categorized as either “reliable with restrictions” or “not reliable,” the two categories which are often the most difficult to assign, and which distinguish between inclusion and exclusion of studies in a hazard or risk assessment. Two studies reported the same experimental data, one in the form of an industry study report (study E) and the other as a peer-reviewed publication (study F).

### Reliability and relevance evaluations

A total of 121 study evaluations were performed by 62 risk assessors in phase I (Klimisch evaluation method), and 104 study evaluations by 54 participants in phase II (CRED evaluation method). Five participants (three in phase I and two in phase II) submitted only one of the two requested questionnaires.

Each study was evaluated by 9–20 risk assessors (Additional file 1: part D, Tables D1, D2), with a mean of 15 per study in phase I and 13 in phase II for both reliability and relevance evaluations. The number of evaluations per study differed because some participants did not submit their completed questionnaires. Reliability and relevance categories assigned to each study were tested for significant differences between evaluation methods using the exact (permutation) version of the Chi-square test in R [37]. False discovery rate was checked according to Benjamini et al. [38]. This is a standard statistical test for categorical data comparison, which evaluates the difference between the numbers of observations (evaluations) in groups of data points (categories).

Arithmetic means and standard deviations of conclusive categories assigned to each study were calculated in order to quantify shifts in evaluation results between methods (Additional file 1: part D, Table D3). Conclusive categories are assigned when a conclusion about the reliability and relevance of a study can be drawn (for reliability: R1, R2, R3; for relevance: C1, C2, C3). Conclusive categories were weighted equally. Non-conclusive categories (R4 and C4) contain those studies where a conclusive category could not be assigned due to a lack of information.

### Consistency analysis

To determine whether one of the tested evaluation methods would produce more consistent results than the other method, the percentage of evaluations in each conclusive reliability (R1–R3) or relevance (C1–C3) category was calculated for each study. The difference between the highest percentage and the average of the two lower percentages was used as a measure of consistency (Additional file 1: part D, Figure D1). For example, 100 % consistency is reached when all participants selected the same evaluation category for a given study. In contrast, if all evaluation categories were selected in equal proportions the consistency is zero. This method was chosen because it neither requires a large number of evaluations per study, nor an equal number of evaluations per evaluation method.

### Practicality analysis

Two main aspects were investigated to compare the practicality of the two evaluation methods: (i) the time required to evaluate a study and (ii) the relationship between the number of fulfilled CRED criteria and assignment of reliability or relevance categories as an indication of the usefulness of the selected criteria for assigning categories. The time required to complete an evaluation using either the Klimisch or CRED evaluation method was reported by participants and compared. Time slots (<20, 20–40, 40–60, 60–180, or >180 min) were chosen to cover a broad temporal scale. Time requirements below 60 min were considered to be indicative of an efficient evaluation system, and this period was therefore examined more closely, hence the choice of smaller time intervals. Results are reported as the percentage of participants per time slot. To determine the relationship between the number of fulfilled CRED criteria and assigned reliability or relevance categories, the number of fulfilled criteria was recorded for each study evaluation. Results are presented by category as arithmetic mean of the percentage of criteria fulfilled, the range, and standard deviation (SD).

### Perception of the evaluation methods

To determine the perception of the two evaluation methods by ring test participants, questionnaires that were completed by risk assessors participating in both phases of the ring test were analyzed. Participants rated their level of agreement with five different statements regarding the accuracy, applicability, consistency, and dependence on expert judgement of each evaluation method. Two additional statements were added during phase II to evaluate the participants’ perception with regard to the transparency of the CRED evaluation method in comparison to the Klimisch method, and to the usefulness of the guidance material provided with the CRED evaluation method (Additional file 1: part D, Table D4). Results were analyzed using the non-parametric pair rank-sum test according to Wilcoxon [39].

Participants were also asked to state the confidence in their evaluation results, ranging from “very confident” to “not confident,” for both evaluation methods using five response alternatives. Data were analyzed using the exact (permutation) version of the Chi-square test in R [40], and checked for false discovery rate according to Benjamini et al. [41]. The percentage of responses was calculated for the Klimisch (n = 121) and CRED evaluation methods (n = 103).

## Results

### Reliability evaluation

**Fig. 1:**
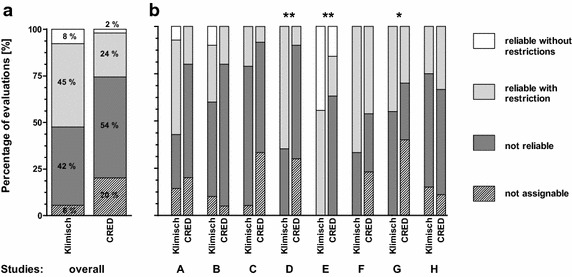
**a** Overall reliability categorization results using the Klimisch (n = 121) and the CRED (n = 104) evaluation methods. **b** Study-specific reliability categories assigned using the Klimisch and CRED evaluation methods for studies A–H. Significant differences were found for studies D and E (**p ≤ 0.01) and study G (*p ≤ 0.05) using the exact (permutation) version of the Chi-square test in R [38]

When using the Klimisch method, 8 % of all evaluations categorized the selected studies as “reliable without restrictions,” 45 % as “reliable with restrictions,” 42 % as “not reliable,” and 6 % as “not assignable.” When using the CRED evaluation method, 2 % of all evaluations categorized the studies as “reliable without restrictions,” 24 % as “reliable with restrictions,” 54 % as “not reliable,” and 20 % as “not assignable” **(**Fig. 1a; Additional file 1: part D, Table D1).

For five of eight studies, reliability categories assigned based on the CRED evaluation method did not differ significantly from those obtained using the Klimisch method (Fig. 1b; Additional file 1: part D Table D1), while significant differences were found for three studies (D, E, G). Two of these (D, E) were categorized as less reliable when using the CRED evaluation method, with the largest difference found for the industry study report (study E). In general, the use of the Klimisch evaluation method resulted in stronger discrepancies among categorizations into the first two (“reliable without restrictions “ and “reliable with restrictions”) versus the last two reliability categories (“not reliable,” “not assignable”) (Fig. 1; Additional file 1: part D, Table D1). For example, studies A, B, and G were evaluated by approximately half of the ring test participants as R1 or R2 (57, 40, and 45 %, respectively), and half as R3 or R4 (43, 60, and 55 %, respectively). This means that the categorization of these studies as regulatory usable or not usable would likely be intensively discussed among experienced risk assessors. In comparison, the use of the CRED evaluation method resulted in studies A, B, and G being assigned to R1 or R2 by only 20 % (studies A and B) and 30 % (study G) of participants, while 80 % (studies A and B) and 70 % (study G) of participants chose categories R3 or R4. A much higher percentage (40 %) of participants chose category “not assignable” (R4) for study G. Similar patterns were observed for studies D and F.

In order to illustrate methodological differences between the two evaluation methods a more detailed analysis of the evaluations for studies D, E, and G, which showed significant differences between evaluation methods, is provided below.

#### Study D [32]

This study reports on the toxicity of three insecticides using a standard algal growth inhibition test with *Desmodesmus subspicatu*s, according to ISO 8692. The risk assessors were asked to evaluate the reliability of a 72-h no observed effect concentration (NOEC) for growth of the insecticide deltamethrin. Using the Klimisch method, 11 of 17 (65 %) ring test participants categorized this study as “reliable with restrictions” and 6 (35 %) as “not reliable.” This was in contrast to the results obtained using the CRED evaluation method which resulted in only 1 of 10 participants (10 %) categorizing the study as “reliable with restrictions,” 6 (60 %) to be “not reliable,” and 3 (30 %) as “not assignable.” The arithmetic means of conclusive categories (R1, R2, R3, not R4) assigned were 2.4 using the Klimisch method and 2.9 using CRED evaluation method (Additional file 1: part D, Table D3).

Independent of the evaluation method used, it was frequently observed by the participants that this study was missing analytical data on exposure concentrations. Of the participants using the Klimisch method, only one participant noted that the exposure concentrations for the test substance (deltamethrin) exceeded its maximum solubility in water; however, still categorized it as “reliable with restrictions.” When the CRED evaluation method was used, nearly all participants who categorized this study as “not reliable” (60 %) listed water exposure concentration above the test substance’s water solubility as one of the reasons. It is surprising that this issue was not detected by participants using the Klimisch evaluation method, since solubility of the test substance is mentioned in both evaluation methods as a factor which may influence test results. We conclude that the more systematic way of performing the evaluations, using a list of criteria (CRED) rather than criteria in text format (Klimisch), may have been sufficient to prompt a more thorough study review regarding this criterion. Thus, important flaws of a study might be more easily discovered if the evaluation is guided by the CRED evaluation method. The additional guidance material provided by the CRED evaluation method [19, 20] might also add to the risk assessor’s confidence when categorizing studies.

#### Study E [33]

This study is an industry study in the form of a GLP report providing fish toxicity data for *Danio rerio* exposed to estrone, a steroidal hormone and metabolite of estradiol. Ring test participants were asked to evaluate the reliability of a 40-day NOEC for sex ratio. Four of nine ring test participants (44 %) using the Klimisch method categorized this study as “reliable without restrictions” and 11 (56 %) as “reliable with restrictions.” With the CRED evaluation method, 3 of 19 participants (16 %) categorized this study as “reliable without restrictions,” 4 (21 %) as “reliable with restrictions,” and the remaining 12 (63 %) as “not reliable.” Independent of the method used, study E was never categorized as “not assignable.” The arithmetic means of conclusive categories (R1–R3) assigned were 1.6 when using the Klimisch method and 2.5 when using the CRED evaluation method (Additional file 1: part D, Table D3).

Using the Klimisch method, some risk assessors remarked that information on test substance purity and solubility as well as raw data in general was missing, yet none of them categorized it as “not reliable” or “not assignable.” In contrast, participants using the CRED evaluation method discovered flaws in the study design related to dosing and potential loss of the test substance. In addition, it was frequently noted that replication and control data provided were insufficient, e.g., due to missing solvent control data. Another issue raised with study E was the uneven number of fish used per treatment group. As for study D, these results suggest that the CRED evaluation method helped risk assessors to detect flaws in study design and reporting.

#### Study G [35]

This study reports fish toxicity data for *Oncorhynchus mykiss* with nonylphenol as a test substance. Participants were asked to evaluate the reliability of a 60-day NOEC for hatching success. This study was categorized as either “reliable with restrictions” by 9 of 20 participants (45 %) or “not reliable” by 11 participants (55 %) using the Klimisch method. Using the CRED evaluation method, it was categorized by 3 of 10 participants (30 %) as “reliable with restrictions,” by 3 (30 %) as “not reliable,” and by 4 (40 %) as “not assignable.” The arithmetic means of conclusive categories (R1–R3) assigned were 2.6 when using the Klimisch method and 2.5 when using the CRED evaluation method (Additional file 1: part D, Table D3).

The main flaw of this study was the use of the solvent dimethylsulfoxide (0.15 %) above the OECD-recommended concentration in test controls and treatments, and the relatively high concentration of 4 % formaldehyde as a disinfectant for fish eggs. Ring test participants using the CRED evaluation method reported additionally that information on the test method was missing, for example, exposure concentrations in the flow through system, purity of the tested substance, and details on feeding of organisms. In this case, the CRED evaluation method appeared to raise awareness regarding the distinction between conclusive (R1–R3) and non-conclusive (R4) categories, the latter referring to the absence of information rather than the inherent quality of the study itself.

### Relevance evaluation

**Fig. 2:**
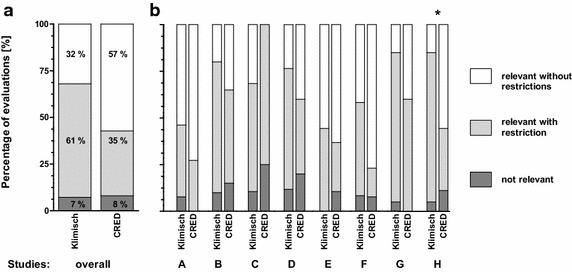
**a** Overall relevance categorization results using the Klimisch (n = 120) and CRED (n = 104) evaluation methods. **b** Study- specific relevance categories assigned using the Klimisch and CRED evaluation methods for studies A–H. Study H differed significantly (*p ≤ 0.05) using the exact (permutation) version of the Chi-square test in R [37]

Overall, the ring test showed that both evaluation methods provide similar results regarding the relevance evaluation of a study, even though a differentiation between “relevant without restrictions” and “relevant with restrictions” is not foreseen in the Klimisch system. Differences occured when deciding whether a study is to be categorized as either “relevant without restrictions” or “relevant with restrictions.” Both the CRED and Klimisch evaluation methods resulted in a high percentage of studies categorized as “relevant without restrictions” (C1) and “relevant with restrictions” (C2) (Fig. 2a; Additional file 1: part D Table D2). Only 7 % of assessors using the Klimisch method and 8 % of assessors using the CRED evaluation method categorized the studies as “not relevant” (C3) for the intended purpose. The proportion of “relevant without restrictions” was 32 % when using the Klimisch and 57 % when using the CRED evaluation method.

For one of the eight studies (study H), results of the relevance evaluations differed significantly between the Klimisch and CRED evaluation methods (Fig. 2b; Additional file 1: part D Table D2) with a higher percentage of studies categorized as “relevant without restrictions” when using the CRED evaluation method. The arithmetic mean of conclusive categories (C1–C3) assigned to this study was 1.9 using the Klimisch method and 1.6 using the CRED evaluation method (Additional file 1: part D Table D3). In line with this evaluation result, 16 of 20 (80 %) ring test participants indicated problems using the Klimisch evaluation method, especially with regard to uncertainties on how to evaluate relevance and the lack of relevance criteria. The fact that study H was not a guideline study was stated more often when the Klimisch evaluation method was used, and more deficiencies of the study were listed. In contrast, 4 of 9 (44 %) participants who used the CRED evaluation method mentioned neither uncertainties nor the lack of criteria as limiting factors. Thus, the use of CRED relevance criteria appeared to result in a lower number of questions regarding the relevance of this study.

### Consistency analysis

The analysis of consistency showed a trend toward more consistency when using the CRED evaluation method. Consistency of reliability categorizations increased by 11–35 % for five of eight studies (A–E), but decreased by 8–12 % for three studies (F–H) (Additional file 1: part D, Fig. D3a). Overall, the average consistency (±SD) of reliability evaluations was 45 % ± 13 (n = 8) when the Klimisch method was used and 56 ± 20 % (n = 8) when the CRED evaluation method was used. For relevance, consistency increased by 3–40 % for six of eight studies (A, C, E–G) when the CRED evaluation method was used, but decreased by 30 and 37 % for studies B and D, respectively (Additional file 1: part D, Fig. D3b). Overall, the average consistency for relevance evaluations was 36 ± 20 % (n = 8) when the Klimisch method was used and 43 ± 20 % (n = 8) when the CRED evaluation method was used.

### Practicality analysis

(i) Time required to evaluate a study: Overall, the two evaluation methods required a similar investment of time per study. Independent of the method used, only a few participants (<10 %) needed less than 20 min (Klimisch: 7 %, CRED: 5 %) or more than 180 min (Klimisch: 2 %, CRED: 3 %) for completing the evaluation. Using the Klimisch method, 68 % of the participants required 20–60 min (20–40 min: 24 %, 40–60 min: 44 %) and 26 % required ≥60 min. When using the CRED evaluation method, 61 % of the participants required 20–60 min (20–40 min: 36 %, 40–60 min: 25 %) and 34 % required ≥60 min.

(ii) Relationship between the number of fulfilled CRED criteria and reliability or relevance categories: The mean number of CRED evaluation criteria fulfilled by a study was associated with the assigned reliability or relevance category (Table 3 and SI part C Fig C2). This indicates that criteria were generally accepted for guiding the assignment of categories (Table 3; Additional file 1: part D Fig D2). The data support the participants’ statements that assignment of critical criteria was not helpful, thus such criteria were not used to categorize studies. On average, when more than 72 % of all criteria were fulfilled, the participants assigned the study to one of the two highest reliability categories with a standard deviation below 15 %; however, the range of percentages of fulfilled criteria for each category can be large. For example, there were evaluations where 90 % of criteria were fulfilled; yet the study was categorized as “not reliable,” and evaluations with 47 % of criteria fulfilled, where the study was categorized as “reliable with restrictions.” For the evaluation of study relevance, on average at least 73 % of criteria had to be fulfilled for the study to be assigned to one of the two highest categories with a standard deviation below 14 %; however, there were evaluations where 82 % of criteria were fulfilled, yet the study was categorized as “not relevant.” These findings suggest that subjectivity may be reduced but is not eliminated when a more detailed assessment system is provided, highlighting the importance of expert judgement.

**Table 3:** Percentage fulfilled criteria for the assigned reliability and relevance categories using the CRED evaluation method

	Percentage of fulfilled criteria^a^				
	Mean	SD	Min	Max	n
Reliability categories					
Reliable without restrictions	93	12	79	100	3
Reliable with restrictions	72	12	47	90	24
Not reliable	60	15	21	90	58
Not assignable	51	15	21	64	19
Relevance categories^b^					
Relevant without restrictions	84	8	64	100	50
Relevant with restrictions	73	14	27	91	42
Not relevant	61	14	46	82	12

Shown are arithmetic mean, standard deviation (SD), minimum and maximum, and the number of evaluations assigned to each reliability and relevance category

^a^The ring test was performed with a draft version of the CRED evaluation method including critical and non-critical criteria; this was not differentiated in this table

^b^The non-conclusive category “not assignable” (C4) is excluded from this analysis, because it was not part of the ring test

### Perception of the evaluation methods

**Fig. 3:**
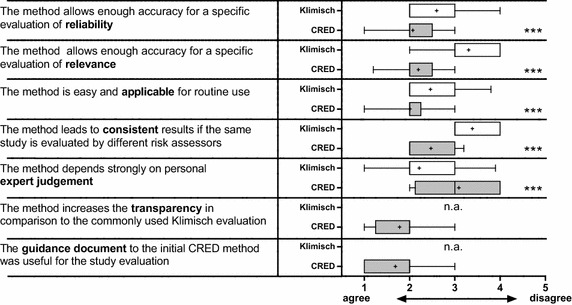
Participants’ (n = 41) agreement regarding the accuracy, applicability, consistency, and dependence on expert judgement for the Klimisch (*white*) and CRED (*gray*) evaluation methods. Results are shown in Whisker plots. *1* totally agree, *2* mainly agree, *3* partially agree, *4* mainly disagree, *5* totally disagree; arithmetic mean of rating is indicated by +. Significant differences (Wilcoxon test) between ratings are indicated by *α < 0.05; **α < 0.01; and ***α < 0.001; *na* not applicable (question was only asked in phase II of the ring test)

**Fig. 4:**
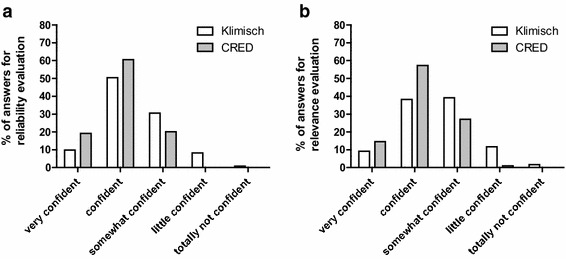
Participants’ confidence in the results of their reliability (**a**) and relevance (**b**) evaluations when using the Klimisch method (n = 121) and the CRED evaluation method (n = 103). Chi-square analysis shows significant differences in the distribution of the responses between the two evaluation methods regarding reliability (p < 0.01) and relevance (p < 0.001)

Analysis of questionnaires showed that participants perceived the CRED evaluation method as significantly more accurate and practical for routine use, as well as less dependent on expert judgement, and was able to produce more consistent results compared to the Klimisch method (Wilcoxon test; p ≤ 0.001) (Fig. 3; Additional file 1: part D Table D4). The majority of the participants “mainly agreed” that the use of the CRED evaluation method improved the transparency of evaluations, and that the guidance material (which was further improved based on feedback and ring test results) provided with each CRED criterion was useful. The participants felt more confident with the results of their evaluation when using the CRED evaluation method (Fig. 4) for both reliability (p < 0.01) and relevance (p < 0.001). For reliability evaluations, 80 % of the participants responded that they felt “very confident” or “confident,” in comparison to 60 % using the Klimisch method. For relevance evaluations, 72 % felt “very confident” or “confident” when using the CRED evaluation method, in comparison to 37 % using the Klimisch method. None of the risk assessors were “little confident” or “totally not confident” in their evaluations when using the CRED evaluation method, compared to 9 % using the Klimisch method.

## Discussion

A thorough and transparent assessment of ecotoxicity studies in regulatory processes is important to ensure that marketing authorizations or decisions on management measures are made on a verifiable basis [9]. The CRED evaluation method [19, 20] was developed to improve the consistency and transparency of study evaluations and to provide guidance for the regulatory use of peer-reviewed aquatic ecotoxicity studies. A draft version of the CRED evaluation method was used in a ring test where selected studies were evaluated, and the results were compared to the evaluations using the Klimisch method. This international ring test involved 12 countries and 35 organizations, including European and North American regulatory agencies, academia, consulting companies, and industry. Participation in the ring test was open to all interested risk and hazard assessors, and due to the participation of a broad range of stakeholders a bias toward specific interest groups is unlikely. In total, 75 risk assessors participated, and approximately 60 % of them had more than 5 years of experience in performing ecotoxicity study evaluations. A ring test of this proportion has, to our knowledge, not been performed before for any of the previously developed evaluation methods for ecotoxicity studies [28].

Our results show that the CRED evaluation method is a suitable and practical replacement for the Klimisch method. It gives more detailed guidance for both reliability and relevance evaluations, which contributed to a greater confidence expressed by the ring test participants regarding their results. In addition, the CRED evaluation method was perceived as more transparent and the resulting categorization as less dependent on expert judgement. Furthermore, the CRED evaluation method was rated to be more practical for routine use than the Klimisch method, while not requiring additional time even though 80 % of participants had prior experience using the Klimisch method.

Increasing the consistency of evaluation results was a goal of the CRED project. Ring test results showed that, overall, consistency of reliability and relevance evaluations increased when the CRED evaluation method was applied. While this was only a trend, this corroborates the participants’ perception that study categorizations based on the CRED evaluation method were more consistent. Additional refinement of the CRED evaluation method into its final version [19, 20], based on the results of the ring test and input from participants, is expected to further increase the consistency of evaluation results. This is particularly relevant in the context of the WFD where EQS for priority substances are to be derived, and where currently considerable differences exist among EQC for river basin-specific pollutants [42, 43]. Inconsistencies in evaluating ecotoxicity studies may contribute to this variability. In general, more consistency in results obtained when using the CRED evaluation method is in line with the participants’ perception of increased accuracy of and confidence in their assessment. This benefits the robustness of an assessment and resulting conclusions drawn by regulators and managers. The CRED evaluation method could, for example, reduce the risk of deriving overprotective PNEC or EQC values resulting in costly risk mitigation measures. At the same time, the increased transparency resulting from the use of the CRED evaluation method ensures that available ecotoxicological evidence cannot be easily dismissed without transparent justification.

Even though the Klimisch method is recommended in many regulatory frameworks, it has been criticized for lack of specific guidance and its focus on standard and guideline tests performed according to GLP [44, 45]. The GLP standard is a quality assurance system regarding technical quality and accuracy of reporting, which does not necessarily guarantee that a study is of sufficient reliability and relevance for regulatory use [46, 47]. When the Klimisch method is used, however, GLP studies may be assigned to a higher reliability category by default [16]. Results of our ring test demonstrate this tendency. Studies E (GLP study report) and F (peer-reviewed publication) report on the same dataset and therefore the categorization results should have been similar. However, categorization results for these two studies were significantly different between the two methods. Using the Klimisch method, the GLP study report was evaluated to be more reliable than the peer-reviewed publication, while an evaluation using the CRED evaluation method resulted in similar categorization of the two studies (Fig. 1b; Additional file 1: part D Table D1).

The CRED evaluation method does not discriminate between peer-reviewed publications and GLP industry study reports because the same reliability and relevance criteria are applied to all studies. This is important as many peer-reviewed publications report on relevant endpoints which are not covered by existing guideline tests. This brings the CRED evaluation method in line with the demands of current regulatory frameworks, such as REACH, WFD, and authorization of plant protection products, biocides, and pharmaceuticals, which recommend the use of all available relevant information. Regarding relevance, ring test results show that the total percentage of studies categorized as “relevant without/with restrictions” did not differ between the CRED and Klimisch evaluation methods. However, more studies were categorized as “relevant without restrictions” when the CRED evaluation method (57 %) than when the Klimisch method (32 %) was used. This may also be due to the more detailed guidance for relevance assessment in the CRED evaluation method [19], thus providing higher certainty for the categorization of studies.

The use of the CRED evaluation method may lead to a more stringent reliability evaluation in some cases. This may be partially related to the differentiation of evaluation criteria into critical and non-critical in the draft method used for the ring test. However, the influence of critical criteria on categorization of studies is expected to be small, as participants rejected their application and felt strongly that expert judgement was needed to decide which criteria were critical based on study design. The inverse relationship between the number of criteria used and assigned categories supports that critical criteria were not or little used. Our results show that when risk assessors use a more systematic evaluation method and are given more detailed and clearer instruction on how to evaluate a study, more flaws in the design, performance, analysis, and reporting of the study are discovered. This method encouraged participants to evaluate a higher number of reliability criteria for both peer-reviewed studies and industry study reports. In the short term, the CRED evaluation method may therefore reduce the amount of studies available for regulatory use. These results should however be regarded with care, as four of the studies (C, F, G, and H) were specifically chosen because they were discussed at the regulatory level as being either “reliable with restrictions” (R2) or “not reliable” (R3). Even small differences between the two evaluation methods are thus expected to have consequences for studies which are not clearly reliable or unreliable, respectively. Nevertheless, it is hoped that the application of CRED reporting recommendations [19] will improve the reliability of studies and therefore, in the long term, increase the number of studies available for regulatory use.

An evaluation method that promotes transparency, such as the CRED evaluation method, can effectively reduce bias related to expert judgment. However, expert judgment should, and cannot, be excluded from the evaluation process. It is needed because the weight of the individual evaluation criteria leading to a final reliability and relevance categorization strongly depends on study design, substance tested, and organisms used. Thus, a single criterion may be critical for one study, but not for another. When a study is evaluated, the final categorization should be based on a balanced assessment of its strengths and weaknesses within a specific framework. Thus, the reliability and relevance categorization of a study should never be based on merely counting the number of fulfilled criteria [48, 49]. Systematic evaluation approaches for toxicological studies such as the ToxRTool [9, 23] emphasize this need for expert judgement. Failure to meet a single criterion considered critical for a particular study result may invalidate a study, while this criterion may not be critical for another study with a different substance or test organism. By requiring the reporting of evaluation results for each criterion, the CRED evaluation method provides documented transparency regarding the use of expert judgment in study evaluations. This allows risk assessors to focus their discussions on the specific strengths and weaknesses of a study rather than solely on Klimisch categories. Such improved transparency can guide and facilitate the identification and use of key studies, and ensure that all relevant and reliable ecotoxicological information is included in future hazard and risk assessment.

## Conclusions and outlook

Hazard and risk assessments in many regulatory frameworks worldwide rely on ecotoxicity studies of sufficient reliability and relevance to allow for the derivation of robust and defensible EQC. The results of the ring test presented here demonstrate that the CRED evaluation method is a suitable and practical method to evaluate the reliability and relevance of aquatic ecotoxicity studies. Its application increases transparency of study evaluations, and can improve the harmonization of risk and hazard assessments, among frameworks as well as countries, institutes, and individual assessors. This can subsequently increase efficiency in the use of resources, since evaluation results from one framework or country can be used more easily in another. This is especially important when EQC are derived for the same compound by different countries or in the context of different legislations. Application of the CRED evaluation method can facilitate the inclusion of relevant peer-reviewed studies in the regulatory process, in accordance with a number of regulatory guidance documents requiring the use of “all available information.” Results of the ring test showed that consistency of assessments only increased to a limited extent; however, it is anticipated that the use of the final method, which was refined based on test results and participant feedback, may further improve this aspect. In the long run, application of the CRED evaluation method, as well as the associated reporting recommendations for peer-reviewed literature, can help increase the availability of relevant and reliable ecotoxicity data needed for regulatory decision making and facilitate the harmonization of regulatory processes. Nevertheless, this new method will need to be applied to a variety of studies before its advantages and limitations will be fully known.

Therefore, the CRED evaluation method is currently piloted and tested in the revision of the EU Technical Guidance Document for EQS values for key studies (P. Whitehouse, Environment Agency, UK, personal communication) and in the revision of EQS proposals for Switzerland. Additionally, the CRED criteria are applied in the Literature Evaluation Tool of the Joint Research Centre (R. Carvalho, JRC, EU Commission, Ispra, Italy, personal communication), as well as in the reliability evaluation of ecotoxicity studies for data bases, such as the NORMAN EMPODAT (P. von der Ohe, Umweltbundesamt, Dessau, Germany, personal communication). In addition, the CRED evaluation method is being considered for inclusion in the project Intelligence-led Assessment of Pharmaceuticals in the Environment (iPiE), which is financed by the pharmaceutical industry and the EU Commission (G. Maack Umweltbundesamt, Dessau, Germany, personal communication).

## Additional file

10.1186/s12302-016-0073-x Supplemental Information (SI): Criteria for Reporting and Evaluating ecotoxicity Data (CRED): Comparison and perception of the Klimisch and CRED methods for evaluating reliability and relevance of ecotoxicity studies
