# Erratum to: Criteria for Reporting and Evaluating ecotoxicity Data (CRED): comparison and perception of the Klimisch and CRED methods for evaluating reliability and relevance of ecotoxicity studies

**DOI:** 10.1186/s12302-016-0079-4

**Published:** 2016-04-11

**Authors:** Robert Kase, Muris Korkaric, Inge Werner, Marlene Ågerstrand

**Affiliations:** 1 Swiss Centre for Applied Ecotoxicology, EAWAG-EPFL, 8600 Dübendorf, Switzerland; 2 Department of Environmental Toxicology, Swiss Federal Institute of Aquatic Science and Technology (Eawag), 8600 Dübendorf, Switzerland; 3 Department of Environmental Science and Analytical Chemistry (ACES), Stockholm University, 106 91 Stockholm, Sweden

## Erratum to: Environ Sci Eur (2016) 28:7 DOI 10.1186/s12302-016-0073-x

After publication of this article [1], the authors noticed an incorrect number was included in the ‘Results’ section of their article under the heading ‘Study E [33]’ [2].

In the original article [1], the number 11 was incorrectly included in the paragraph before “(56 %)”. The correct paragraph for ‘Study E [33]’ [2] including the correct number, “five”, is included in this erratum.

“This study is an industry study in the form of a GLP report providing fish toxicity data for *Danio rerio* exposed to estrone, a steroidal hormone and metabolite of estradiol. Ring test participants were asked to evaluate the reliability of a 40-day NOEC for sex ratio. Four of nine ring test participants (44 %) using the Klimisch method categorized this study as “reliable without restrictions” and five (56 %) as “reliable with restrictions.” With the CRED evaluation method, 3 of 19 participants (16 %) categorized this study as “reliable without restrictions,” 4 (21 %) as “reliable with restrictions,” and the remaining 12 (63 %) as “not reliable.” Independent of the method used, study E was never categorized as “not assignable.” The arithmetic means of conclusive categories (R1–R3) assigned were 1.6 when using the Klimisch method and 2.5 when using the CRED evaluation method (Additional file 1: part D, Table D3).”
