# Supplementary material for: Criteria for Reporting and Evaluating ecotoxicity Data (CRED): comparison and perception of the Klimisch and CRED methods for evaluating reliability and relevance of ecotoxicity studies
Source: Environ Sci Eur. 2016 Feb 29;28(1):7. doi: 10.1186/s12302-016-0073-x (PMC5044958; doi:10.1186/s12302-016-0073-x)
Supplement: Supplementary file 1 — 10.1186/s12302-016-0073-x Supplemental Information (SI): Criteria for Reporting and Evaluating ecotoxicity Data (CRED): Comparison and perception of the Klimisch and CRED methods for evaluating reliability and relevance of ecotoxicity studies [file 12302_2016_73_MOESM1_ESM.pdf]

Supplemental Information (SI): Criteria for Reporting and Evaluating ecotoxicity Data (CRED): Comparison and perception of the Klimisch and CRED methods for evaluating reliability and relevance of ecotoxicity studies

Part A:

Example of a Klimisch evaluation criteria and ring test questionnaire (Phase I)

PERSONAL INFORMATION

Name and affiliation

BEFORE YOU START

- If you are not familiar with the Klimisch assessment, please read the ***Klimisch et al. 1997*** publication in the annex of your e-mail.
- For this study evaluation, please **only** focus on the ***substance, species*** *and* ***endpoint*** listed below in study information.

STUDY INFORMATION

Please analyse the following study concerning:

Study	**Lambert et al. 2006**
Study number	No. 1

Substance class	Biocide
Substance	**Cybutryne**
Taxonomic group	Macrophytes
Species	***Myriophyllum spicatum***
Endpoint	**NOEC 14 d growth / biomass**

EVALUATION OF RELIABILITY (SEE KLIMISCH ET AL. 1997, ANNEX OF YOUR E-MAIL)

Please indicate the reliability of the presented study for the defined substance, species and endpoint by assigning one of the 4 Klimisch-codes (Please mark “X")

Klimisch code	Category	**Your assignment**	With tendency towards (if applicable)
1	Reliable without restrictions		
2	Reliable with restrictions		
3	Not reliable		
4	Not assignable		

QUESTIONS REGARDING YOUR RELIABILITY EVALUATION

How safe did you feel in your evaluation with the Klimisch approach, regarding **reliability**?	Very safe	Safe	Somewhat safe	Unsafe	Totally unsafe

Please state in a few words up to three major uncertainties in your study evaluation regarding **reliability**?<br>Did you find missing **reliability criteria** for an accurate study evaluation?	
**Uncertainties regarding reliability**	**Missing reliability criteria**

From your point of view, how could the presented study improve its **reliability**?

QUESTIONS REGARDING STUDY RELEVANCE

Although the *relevance* aspect is mentioned in Klimisch et al .1997, a specific classification scheme for relevance is not described. However, according to REACh and WFD (TGD for EQS) and other regulations the relevance aspect is important for a hazard assessment. Therefore we would like to ask for your relevance estimation for the presented study (using the aspects mentioned in Klimisch et al. 1997 and your own experience).

Please indicate your estimation of the **relevance** of the presented study for the defined substance, species and endpoint.	Relevant without restrictions	Relevant with restrictions	Not relevant

How safe did you feel in your evaluation with the Klimisch approach, regarding **relevance**?	Very safe	Safe	Somewhat safe	Unsafe	Totally unsafe

Please state in a few words up to three major uncertainties in your study evaluation regarding **relevance**?<br>If you are not sure on **relevance,** what kind of relevance criteria would be helpful to you?	
**Uncertainties regarding relevance**	**Identified missing relevance aspects**

From your point of view, how could the presented study improve its **relevance**?

SPECIFIC QUESTIONS REGARDING THE STUDY QUALITY

How easy was it for you to extract information from the presented material which was necessary for the evaluation?	Very easy	Easy	OK	Difficult	Very difficult

What was your impression of the **general quality** of the study in terms of **aquatic ecotox data**?	Very good	Good	OK	Bad	Very bad

What was your impression of the **general quality** of the study in terms of **structure and clarity of writing**?	Very good	Good	OK	Bad	Very bad

Please describe the strengths (maximum three) of the study in catchwords:

TIME REQUIREMENT AND YOUR EXPERIENCE LEVEL

How long did it take you to evaluate the study (without studying the additional material like the Klimisch paper)?	<20 min	20-40 min	40-60 min	60-180 min	>180 min

How much experience in aquatic ecotox study evaluations do you have (in years)?	0-1	1-2	2-5	5-10	>10 years

Have you ever used the current **Klimisch approach** to evaluate a study?	**YES**		**NO**		
**Please indicate your agreement with the following statements**	Totally agree	Mainly agree	Partially agree	Mainly disagree	Totally disagree
The current Klimisch evaluation allows enough accuracy for a specific evaluation of **reliability**.					
The current Klimisch evaluation allows enough accuracy for a specific evaluation of **relevance.**					

The Klimisch evaluation is **easy** and **applicable** for routine use.					
The use of the current Klimisch evaluation leads to **consistent results** if the same study is evaluated by different risk assessors.					
The current Klimisch evaluation depends strongly on **personal expert judgement.**					

Part B:

Example of a CRED evaluation criteria and ring test questionnaire (phase II)

PERSONAL INFORMATION

Name and affiliation

BEFORE YOU START

- Please familiarize yourself with the guidance document for our checklist for reliability and relevance evaluation in the annex of your e-mail.
- For this study evaluation, please <u>**only**</u> focus on the ***substance, species*** *and* ***endpoint*** listed below in study information.
- Please keep track of the time you needed to evaluate the current study.

STUDY INFORMATION

Please analyse the following study concerning

Study	**Lambert et al. 2006**
Study number	No. 1

Substance class	Biocide
Substance	**Cybutryne**
Taxonomic group	Macrophytes
Species	***Myriophyllum spicatum***
Endpoint	**NOEC 14 d growth / biomass**

The study is evaluated for the purpose of deriving a risk limit for freshwater (e.g., a PNEC or EQS).

Study Evaluation with Reliability Checklist

Short guidance for evaluating checklist results (for an explanation of the criteria, please read the accompanying document)

1) If a critical criterion is not fulfilled, the study reliability decreases directly to reliability score 3. If the information available does not make an assessment of the critical criteria possible, the reliability score 4.
2) If one or several non-critical criteria are not fulfilled or not applicable, expert judgment on reliability is necessary to decide between reliability score of 2 and 3.
3) This checklist is a selection of important criteria for most aquatic ecotoxicity studies. If more detailed criteria are deemed to be necessary to assess a publication, please refer to Agerstrand et al. 2011, where a longer checklist of 62 criteria is available, or to Mensink et al., 2008 (see text document for references).

Nr.	Reliability Check list	Critical	Your evaluation		
	**General information**		Please mark your answer with **x** and only give **one x** per criterion		
	Before evaluating the test, check the physicochemical characteristics of your compound (handbooks/general sources). What is the solubility, log KOW, pKa, is the compound volatile, does it hydrolyse, photolyse, etc.?	not applicable	**Criterion fulfilled**	**Criterion not fulfilled**	**Criterion not applicable for this study**
**1**	Is a description of endpoints and methodology available?	**Yes**			
	**Protocol**				
**2**	Is a standard method (e.g., OECD/ISO) or modified standard used?	**No**			
**3**	Is the test performed under GLP conditions?	**No**			
**4**	If applicable, are validity criteria fulfilled (e.g. control survival, growth)?	**Yes.** Criteria depend on test organism			
**5**	Are appropriate controls performed (e.g. solvent control, negative and positive control)?	**Yes.** Type of control depends on test substance & protocol			
	**Test Compound**				
**6**	Is the tested substance identified clearly with name or CAS-number? Are test results reported for the appropriate compound?	**Yes**			
**7**	Is the purity or the source reported, is there information on the formulation available (if appropriate)?	**Yes**			
	**Test Organism**				
**8**	Are the organisms well described (e.g. scientific name, weight, length, growth, age/life stage,	**Yes.** Necessary details depend			

	strain/clone)?	on test organism			
**9**	Are the test organisms from a trustworthy source and acclimatized to test conditions? Have the organisms not been pre-exposed to test compound or other unintended stressors?	**Yes**			
	**Exposure Conditions**				
**10**	Is the experimental system appropriate for the test substance and are appropriate test vessels used (e.g. , static, flow-through, renewal; light/dark conditions; open/closed systems)?	**Yes**			
**11**	Is the experimental system appropriate for the test organism; e.g., choice of medium or test water, feeding, water characteristics, temperature, light/dark conditions, pH, oxygen content?	**No**. Exceptions possible			
**12**	Do the exposure concentrations not exceed water solubility? Or, if a solvent is used, is the solvent within the appropriate range and is a solvent control included?	**Yes**			
**13**	Is a correct spacing between exposure concentrations applied (e.g. , maximum factor of 10, OECD recommends a factor of 3.2)?	**Yes**			
**14**	Have chemical analyses been performed to verify substance concentrations?	**Yes.** Exceptions possible			
**15**	Is the loading of the organisms within the appropriate range (< 1 g/L)?	**Yes**, for hydrophobic compounds			
	**Statistical Design and Biological Response**				
**16**	Is there a sufficient number of replicates, and a sufficient number of organisms per replicate for all controls and test concentrations?	**Yes**			
**17**	Are appropriate statistical methods used?	**Yes.** Can also be recalculated by assessor afterwards if enough information is provided			
**18**	Is a dose response curve observed? Is the response statistically significant?	**Yes.** Can also be recalculated by assessor afterwards if enough information is provided			
**19**	Are raw data available?	**No** (not critical for reliability 2, but essential for reliability 1)			

NB: The final CRED evaluation and reporting method, including an excel sheet for practical use is available in Moermond et al. 2015 [1] and can be downloaded at: http://www.ecotoxcentre.ch/projects/risk-assessment/cred/

Final reliability assessment after reliability check

Please indicate the reliability of the presented study for the defined substance, species and endpoint by assigning one of the 4 Reliability-scores (Please mark "**X**")

Reliability (Ri) scores	Category	**Your assignment**	With tendency towards (if applicable)
1	Reliable without restrictions		
2	Reliable with restrictions		
3	Not reliable		
4	Not assignable		

We used the Reliability scores 1 -4, based on the original Klimisch codes. What is your opinion on a scoring system where the reliability scores 1 and 2 are combined? (e.g., Ri 1 = reliable; Ri3 = not reliable; Ri 4 = not assignable).		
I'd prefer Reliability scores 1-4	I'd prefer Reliability scores 1-3	comments

In the framework where you work, is a distinction made between the Klimisch codes 1 and 2? Are Klimisch codes 1 and 2 used with equal weight in risk assessment or risk limit derivation?

QUESTIONS REGARDING YOUR RELIABILITY EVALUATION

How safe did you feel in your evaluation with the current approach, regarding **reliability**?	Very safe	Safe	Somewhat safe	Unsafe	Totally unsafe

For further improvement, please characterize our **reliability** checklist approach after your study evaluation	
**Did you find unclear criteria?** If yes, please specify below.	**Did you find missing criteria?** If yes, please specify below.

Please comment how the presented study could best improve its **reliability**?

Nr.	Relevance Check list	Critical	Importance weighting			Your evaluation		
	**General**							
	Before evaluating the test for relevance, check why you are evaluating this study. The relevance of the study might be different for different purposes (e.g., EQS derivation, PBT assessment, dossier evaluation for marketing authorisation), also depending on the framework for which the evaluation is requested. **(For this ringtest, please consider the study to be evaluated for the purpose of deriving a risk limit for freshwater; for instance a PNEC or EQS).**		Please weight criteria according to given importance			Please mark your answer with **x** and only give **one x** per criterion		
			**High**	**Medium**	**low**	**Criterion fulfilled**	**Not fulfilled**	**Not applicable for this study**
	**Biological relevance**							
**1**	Is the species tested relevant for the aquatic compartment and the tested compound?	**Yes**						
**2**	Are the reported endpoints appropriate for the investigated effects or the mode of action?	**Yes**						
**3**	Is the effect population relevant?	**Yes**. Depends on framework						
**4**	Is the magnitude of effect (e.g. EC5, EC10, EC50) relevant according to the guideline?	**Yes**						
**5**	Are appropriate life-stages studied?	**Yes**						
**6**	Are the experimental conditions relevant for the tested species?	**Yes**						
**7**	Is the time of exposure relevant and appropriate for the studied endpoints and species?	**Yes**						
**8**	If recovery is studied, is this relevant for the framework for which the study is evaluated?	Depends on framework						
	**Exposure relevance**							
**9**	Is the substance tested representative and relevant for the substance being assessed?	**Yes**						
**10**	Is the tested exposure scenario relevant for the substance?	**Yes**						
**11**	Do the tested concentrations relate to measured or predicted environmental concentrations (if available)	**No**						

NB: The final CRED evaluation and reporting method, including an excel sheet for practical use is available in Moermond et al. 2015 [1] and can be downloaded at: http://www.ecotoxcentre.ch/projects/risk-assessment/cred/ [2]

Final relevance assessment after relevance checklist

Please indicate the **relevance** of the presented study for the defined substance, species and endpoint by assigning one of the 3 Relevance-codes (Please mark “**x**")

Relevance class	Category	**Your assignment**
1	Relevant without restrictions	
2	Relevant with restrictions	
3	Not relevant	

Above, we have used the Relevance scores 1 -3 as in Phase I of the ringtest. However, other scoring systems are also possible. Which of the following **relevance** scoring systems do you think are appropriate/practical? (indicate with **x**)		
	a) Two classes with …	1. Relevant<br>3. Not relevant
	b) Three classes with …	1. Relevant without restrictions<br>2. Relevant with restrictions<br>3. Not relevant
	c) Three classes with …	1. Relevant without restrictions<br>3. Not relevant<br>4. Not assignable
	d) Four classes with …	1. Relevant without restrictions<br>2. Relevant with restrictions<br>3. Not relevant<br>4. Not assignable

How safe did you feel in your evaluation with the current approach, regarding **relevance**?	Very safe	Safe	Somewhat safe	Unsafe	Totally unsafe

For further improvement, please characterize our **relevance** checklist approach after your study evaluation	
**Did you find unclear criteria? If yes, please specify below.**	**Did you find missing criteria? If yes, please specify below.**

Please comment how the presented study could best improve its **relevance**?

SPECIFIC QUESTIONS REGARDING THE EVALUATED STUDY

How easy was it for you to extract information from the presented material which was necessary for the evaluation?	Very easy	Easy	Fair	Difficult	Very difficult

What was your impression of the **general quality** of the study in terms of **aquatic ecotox data**?	Very good	Good	Fair	Bad	Very bad

What was your impression of the **general quality** of the study in terms of **structure and clarity of writing**?	Very good	Good	Fair	Bad	Very bad

Please describe the strengths (maximum three) of the study in catchwords:

TIME REQUIREMENT AND YOUR EXPERIENCE LEVEL

How long did it take you to evaluate the study (without studying the additional material like the checklist)?	<20 min	20-40 min	40-60 min	60-180 min	>180 min

How much experience in aquatic ecotox study evaluations do you have (in years)?	0-1	1-2	2-5	5-10	>10 years

**Please indicate your agreement with the following statements**	Totally agree	Mainly agree	Partially agree	Mainly disagree	Totally disagree
• The checklist approach allows enough accuracy for a specific evaluation of **reliability**.					
• The checklist approach allows enough accuracy for a specific evaluation of **relevance.**					
• The checklist approach is **easy** and **applicable** for routine use.					
• The use of the checklist approach leads to **consistent results** if the same study is evaluated by different risk assessors.					
• The checklist approach depends strongly on **personal expert judgement.**					
• The checklist approach increases the **transparency** in comparison to the commonly used Klimisch evaluation.					
• The **guidance document** to the checklists was useful for the study evaluation					

NB: The final CRED evaluation and reporting method, including an excel sheet for practical use is available in Moermond et al. 2015 [1] and can be downloaded at: http://www.ecotoxcentre.ch/projects/risk-assessment/cred/ [2]

Part C:

Changes of the draft CRED to the final CRED evaluation method

Table C1: Summary of changes between the initially tested draft CRED evaluation criteria and the final CRED Moermond et al. 2015 [1] evaluation criteria. Differences are highlighted and general comments in italics.

**Changes**	**Draft CRED method**	**Final CRED method**
Number of reliability criteria	19	20
Number of relevance criteria	11	13
Suggested use of critical criteria	yes	no
Justification of evaluation by criterion	no	yes, for fulfillment and not fulfillment
Response options for each criterion	3<br>(fulfilled, not fulfilled, not applicable for this study)	4<br>(fulfilled, not fulfilled, not applicable, not reported)
Weighting of criteria	only asked for the relevance criteria[#]	no
**Modifications to reliability criteria; criteria numbers correspond to criteria used by [1]**	**Draft CRED method**	**Final CRED method**

No. 1	Is a **standard** method (e.g., OECD/ISO) or modified standard used?	Is a guideline method (e.g., OECD/ISO) or modified guideline used?*<br>*These criteria are of minor importance for study reliability but may support study evaluation
No. 2	Is the test performed under GLP conditions?	Is the test performed under GLP conditions? *<br>*These criteria are of minor importance for study reliability but may support study evaluation
No. 6	Is the purity or the source reported, **is there information on the formulation available (if appropriate)?**	Is the purity of the test substance reported? Or, is the source of the test substance trustworthy?
No. 7	*criterion was not available in the draft version*	If a formulation is used or if impurities are present: Do other ingredients in the formulation exert an effect? Is the amount of test substance in the formulation known?
No 8	Are the organisms well described (e.g. scientific name, weight, length, growth, age/life stage, strain/clone)?	Are the organisms well described (e.g. scientific name, weight, length, growth, age/life stage, strain/clone, **gender if appropriate**)?
No.10	Is the experimental system appropriate for the test substance **and are appropriate test vessels used (e.g. , static, flow-through, renewal; light/dark conditions; open/closed systems)?**	Is the experimental system appropriate for the test substance, taking into account its physicochemical characteristics?
No. 11	Is the experimental system appropriate for the test organism; e.g., choice of medium or test water, feeding, water characteristics, temperature, light/dark conditions, pH, oxygen content?	Is the experimental system appropriate for the test organism (e.g., choice of medium or test water, feeding, water characteristics, temperature, light/dark conditions, pH, oxygen content)? **Have conditions been stable during the test?**
No. 12	Do the exposure concentrations not exceed water solubility? Or, if a solvent is used, is the solvent within the appropriate range and is a solvent control included?	**Were exposure concentrations below the limit of water solubility (taking the use of a solvent into account)?** If a solvent is used, is the solvent within the appropriate range and is a solvent control included?

No. 13	Is a correct spacing between exposure concentrations applied **(e.g. , maximum factor of 10, OECD recommends a factor of 3.2)**?	Is a correct spacing between exposure concentrations applied?
No. 14	*criterion was not available in the draft version*	Is the exposure duration defined?
No. 15	Have chemical analyses been performed to verify substance concentrations?	Are chemical analyses adequate to verify concentrations of the test substance **over the duration of the study?**
No.16	Is the loading of the organisms in the test system within the appropriate range (e.g. < 1 g/L)?	Is the **biomass** loading of the organisms in the test system within the appropriate range (e.g. < 1 g/L)?
No. 17	Is **there** a sufficient number of replicates, **and** a sufficient number of organisms per replicate for all controls and test concentrations?	Is a sufficient number of replicates used? Is a sufficient number of organisms per replicate used for all controls and test concentrations?
No. 19	Is a **dose** response curve observed? Is the response statistically significant?	Is a concentration-response curve observed? Is the response statistically significant?
No. 20	Are raw data available?	Are **sufficient data available to check the calculation of endpoints** and (if applicable) validity criteria (e.g., control data, concentration-response curves)?
**Modifications to relevance criteria; criteria numbers correspond to criteria used by [1]**	**Draft CRED method**	**Final CRED method**
No. 1	Is the species tested relevant for the aquatic compartment **and the tested compound?**	Is the species tested relevant for the compartment under evaluation?
No. 2	*see criterion above*	Are the organisms tested relevant for the tested compound?

No. 3	Is the effect population relevant?	Are the reported endpoints appropriate for the regulatory purpose?
No. 4	Are the reported endpoints appropriate for the investigated effects or the mode of action?	Are the reported endpoints appropriate for the investigated effects or the mode of action **of the test substance**?
No. 5	Is the effect population relevant?	Is the effect relevant on a population level?
No. 6	Is the magnitude of effect (e.g. EC5, EC10, EC50) relevant according to the guideline?	Is the magnitude of effect **statistically significant** and **biologically** relevant for **the regulatory purpose** (e.g. EC10, EC50)?
No. 9	Is the **time of exposure** relevant and appropriate for the studied endpoints and species?	Is the **exposure duration** relevant and appropriate for the studied endpoints and species?
No.11	Is the substance tested representative and relevant for the substance being assessed?	**In case of a formulation, other mixture, salts or transformation products:** Is the substance tested representative and relevant for the substance being assessed?
No. 13	*criterion was not available in the draft version*	Is the tested exposure scenario relevant for the species?

this aspect was not completed by participants

Part D:

Ring test results and data analysis

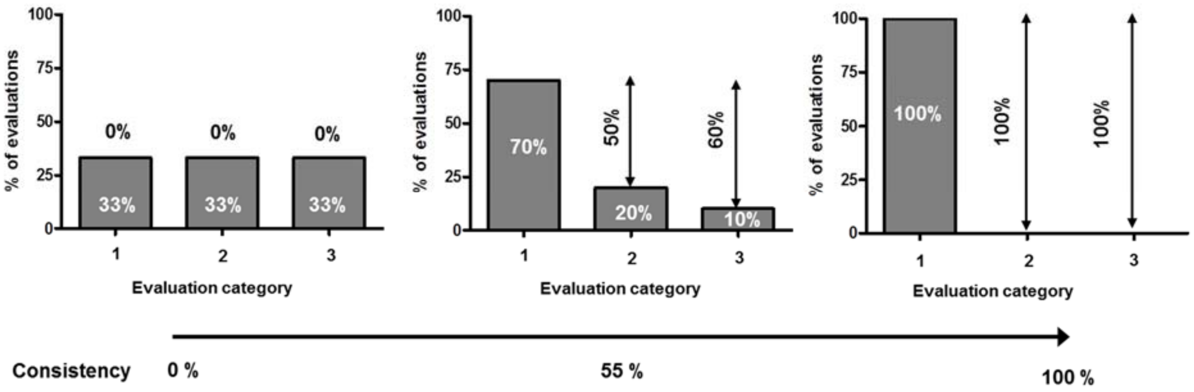

Figure D1: Examples for the principle of the consistency analysis Consistency was measured as the average distance to the most frequently selected evaluation category for reliability (R) or for relevance (C).

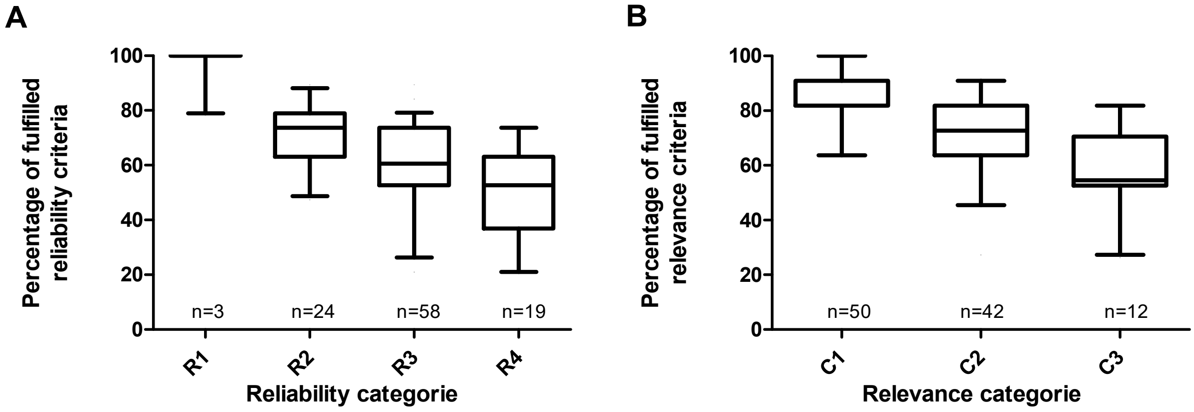

Figure D2: Percentages of fulfilled CRED reliability criteria (**A**) and relevance criteria (**B**) for each study category (n=104). Results are shown with Whisker Plots and median. R1 = reliable without restrictions, R2 = reliable with restrictions, R3 = not reliable, R4 = not assignable, C1= relevant without restrictions, C2 = relevant with restrictions, C3 = not relevant.

A

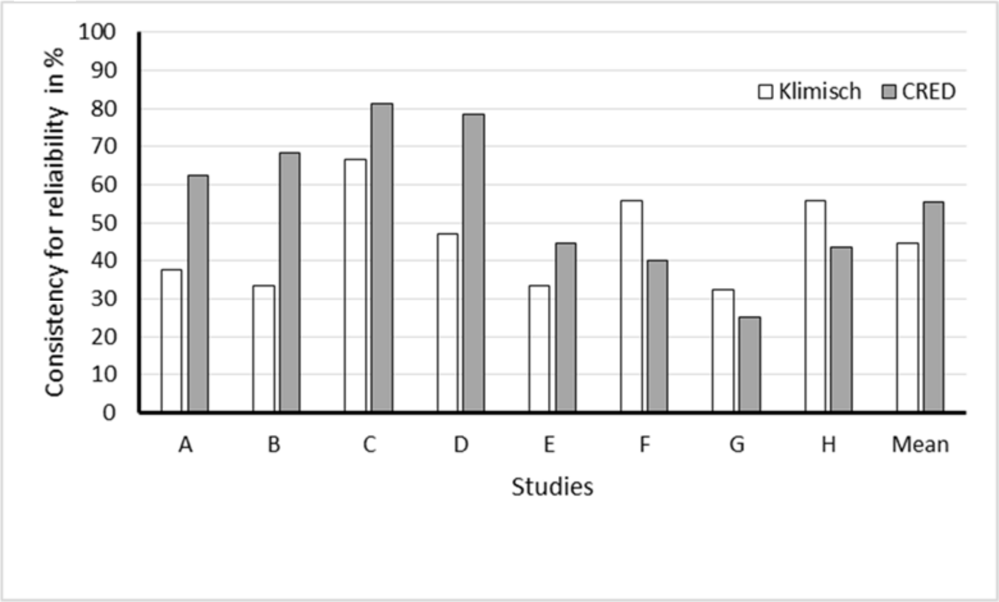

B

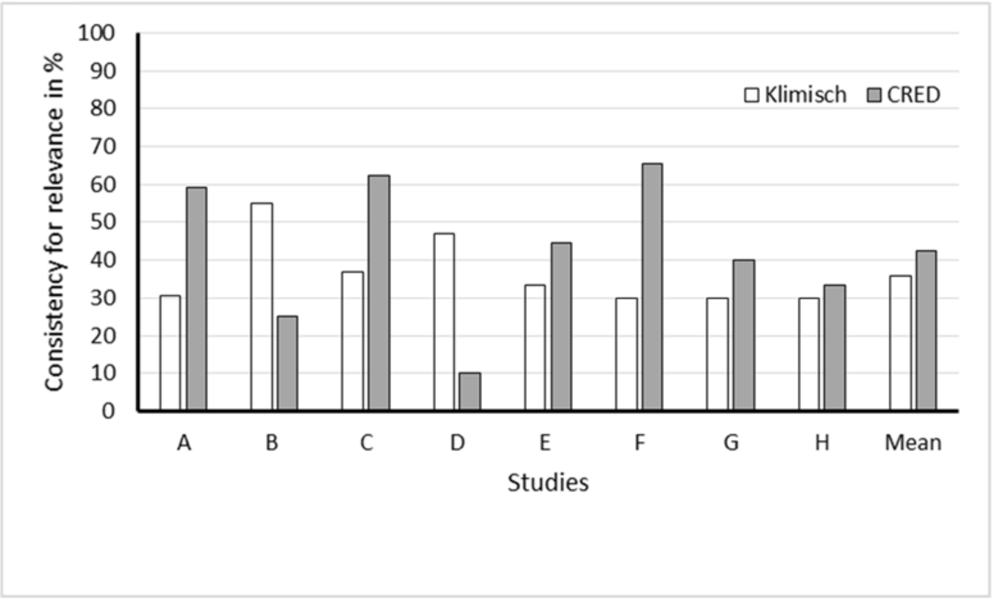

Figure D3: Consistency of reliability (A) and relevance (B) evaluation results for the Klimisch and CRED evaluation methods (R1, R2, R3, not R4, and C1, C2, C3). Mean consistency of reliability evaluations was 45% ± 12% in phase I (Klimisch) and 56%± 20% in phase II (CRED); mean consistency of relevance evaluations was 36% ± 10% in phase I (Klimisch) and 43% ± 19% in phase II (CRED).

Table D1: Results of reliability evaluations using the Klimisch and CRED evaluation methods

	Number of evaluations per study		p-value[a]	Reliability categorisations with Klimisch in %				Reliability categorisations with CRED in %			
Study	Klimisch	CRED		R1	R2	R3	R4	R1	R2	R3	R4
A	14	10	0.331	7	50	29	14	0	20	60	20
B	10	20	0.457	10	30	50	10	0	20	75	5
C	19	12	0.108	0	21	74	5	0	8	58	33
D	17	10	0.004	0	65	35	0	0	10	60	30
E	9	19	0.005	44	56	0	0	16	21	63	0
F	12	13	0.321	0	67	33	0	0	46	31	23
G	20	10	0.013	0	45	55	0	0	30	30	40
H	20	9	1.000	0	25	60	15	0	33	56	11
**Mean**	**15.1**	**12.9**									

[a] p-values for the difference between Klimisch and CRED categorizations, calculated using the chi-square test.

Table D2: Results of relevance evaluations using the Klimisch and CRED evaluation methods

	Number of evaluations per study		p-value[a]	Percentage of relevance categorisations with Klimisch in %			Percentage of relevance categorisations with CRED in %		
Study	Klimisch	CRED		C1	C2	C3	C1	C2	C3
A	13	11	0.821	54	38	8	73	27	0
B	10	20	0.565	20	70	10	35	50	15
C	19	12	0.050	32	58	11	0	75	25
D	17	10	0.463	24	65	12	40	40	20
E	9	19	0.484	56	44	0	63	26	11
F	12	13	0.179	42	50	8	77	15	8
G	20	10	0.341	15	80	5	40	60	0
H	20	9	0.037	15	80	5	56	33	11
**Mean**	**15.0**	**13.0**							

[a] p-values for the difference between Klimisch and CRED categorizations, calculated using the chi-square test.

Table D3: Results of reliability and relevance ring test evaluations using the Klimisch and CRED evaluation methods. Arithmetic means and standard deviations of conclusive categories R1, R2, R3, and C1, C2, C3 (weighted numerically equally as 1,2,3) assigned to each study. Higher means indicate lower reliability or relevance; lower means indicate higher reliability or relevance.

	Arithmetic mean ± SD of reliability categories R1-R3 (% evaluations in categories R1-R3)[a]		Arithmetic mean ± SD of relevance evaluation categories C1-C3[b]	
Study	Klimisch	CRED	Klimisch	CRED
A	2.3 ± 0.6 (86)	2.3 ± 0.6 (80)	1.5 ± 0.7	1.3 ± 0.5
B	2.4 ± 0.7 (90)	2.8 ± 0.5 (95)	1.9 ± 0.6	1.8 ± 0.7
C	2.8 ± 0.4 (95)	2.4 ± 0.7 (66)	1.8 ± 0.6	2.3 ± 0.5
D	2.4 ± 0.5 (100)	2.8 ± 0.4 (70)	1.9 ± 0.6	1.8 ± 0.8
E	1.6 ± 0.5 (100)	2.8 ± 0.4 (100)	1.4 ± 0.5	1.5 ± 0.7
F	2.3 ± 0.5 (100)	2.9 ± 0.4 (67)	1.7 ± 0.6	1.3 ± 0.6
G	2.6 ± 0.5 (100)	2.4 ± 0.5 (60)	1.9 ± 0.5	1.6 ± 0.5
H	2.7 ± 0.5 (85)	2.9 ± 0.4 (89)	1.9 ± 0.5	1.6 ± 0.7

[a] “reliable without restrictions” (R1), “reliable with restrictions” (R2), and “not reliable” (R3).

[b] “relevant without restrictions” (C1), “relevant with restrictions” (C2), and “not relevant” (C3)

Table D4: Ring test participants average perception of the Klimisch and CRED evaluation methods. The rankings (1 = totally agree, 2 = mainly agree, 3 = partially agree, 4 = mainly disagree, 5 = totally disagree) were used to calculate a mean answer. Data were analyzed using the Wilcoxon ranking test for pairs (n=41); lowest ranking sum, for α=0.05=>lower 249, α=0.01=> lower 207, α=0.001=>lower 161; SD = standard deviation; questions 6 and 7 were only asked after use of the CRED evaluation method.

**Questionnaire Statement**	**Klimisch mean ± SD**	**CRED mean ± SD**	**Lowest ranking sum**	**p-value**
1) The evaluation method allows enough accuracy for a specific evaluation of reliability.	2.60 ± 0.77	2.07 ± 0.63	59.5	<0.001
2) The evaluation method allows enough accuracy for a specific evaluation of relevance.	3.32 ± 0.76	2.20 ± 0.62	17	<0.001
3) The evaluation method is easy and applicable for routine use.	2.51 ± 0.78	2.01 ± 0.66	31.5	<0.001
4) The evaluation method leads to consistent results if the same study is evaluated by different risk assessors.	3.44 ± 0.73	2.47 ± 0.76	20.5	<0.001
5) The evaluation method depends strongly on expert judgement.	2.13 ± 0.83	3.09 ± 0.80	55.5	<0.001
6) The CRED evaluation method increases the transparency in comparison to the Klimisch method.	NA	1.78 ± 0.58 (n=54)		
7) The CRED guidance document was useful for study evaluation	NA	1.66 ± 0.62 (n=53)		

References

[1] Moermond CTA, Kase R, Korkaric M, Ågerstrand M 2015. CRED: Criteria for Reporting and Evaluating ecotoxicity Data. Environmental Toxicology and Chemistry. Open access. Accepted on 24 September 2015. DOI: 10.1002/etc.3259. Available at: http://onlinelibrary.wiley.com/doi/10.1002/etc.3259/pdf. Accessed 27 October 2015

[2] Swiss Centre for Applied Ecotoxicology Eawag-EPFL: CRED evaluation excel tool for reliability and relevance. 2015. Available at: http://www.ecotoxcentre.ch/projects/risk-assessment/cred/. Accessed at 11 November 2015
